# Outcome of a single XEN microstent implant for glaucoma patients with different types of glaucoma

**DOI:** 10.1186/s12886-020-01764-8

**Published:** 2020-12-17

**Authors:** Marc Schargus, Theresa Theilig, Matus Rehak, Catharina Busch, Caroline Bormann, Jan Darius Unterlauft

**Affiliations:** 1 Department of Ophthalmology, Heinrich-Heine-University, Duesseldorf, Germany; 2 Department of Ophthalmology, University Eye Hospital, University of Leipzig, Liebigstrasse 10-14, 04103 Leipzig, Germany

**Keywords:** Primary open-angle Glaucoma, Trabeculectomy, XEN, MIGS

## Abstract

**Background:**

The aim of this retrospective study was to compare the efficacy and safety profile of a single XEN-microstent in different types of primary and secondary open angle glaucoma.

**Methods:**

A single XEN microstent was implanted in patients with primary open-angle glaucoma (POAG), normal-tension glaucoma (NTG), pseudoexfoliation glaucoma (PEX) and secondary glaucoma (Sec.Gl). The intraocular pressure (IOP), the active substances of the applied IOP-lowering drugs, the best corrected visual acuity (BCVA) and the mean deviation (MD) of the perimetry were measured at baseline and at regular follow-ups, scheduled at 2 days and 1, 3, 6 and 12 months after surgery.

**Results:**

153 eyes were included in this analysis. 113 eyes were affected by POAG (74%), 5 eyes by NTG (3%), 22 eyes by PEX (14%) and 13 eyes by Sec. Gl (9%). Mean IOP decreased in all treatment groups during the 12 months of follow-up (complete group: 23.9 ± 7.4 to 15.4 ± 5.1 mmHg (*p* < 0.01); POAG: 22.8 ± 6.5 to 15.1 ± 4.6 mmHg (*p* < 0.01); NTG: 16.6 ± 3.4 to 11.6 ± 2.2 mmHg (*p* < 0.05); PEX: 28.0 ± 7.9 to 17.1 ± 6.6 mmHg (*p <* 0.01); Sec.Gl: 28.9 ± 13.9 to 15.5 ± 6.9 mmHg (*p <* 0.05)). In the 153 eyes the average number of IOP-lowering drugs applied decreased from 2.6 ± 1.2 to 0.8 ± 1.3 12 months after surgery (*p* < 0.01). BCVA and mean deviation of automated standard perimetry remained stable in all groups during follow-up.

**Conclusion:**

As in eyes suffering from POAG, IOP and number of IOP-lowering drugs applied can be effectively reduced by XEN implantation in eyes suffering from NTG, PEX and secondary glaucoma while leaving BCVA and visual field unchanged.

**Trial registration:**

Trial was registered at DRKS (registration number: DRKS00020800, Registered 25.February 2020 - Retrospectively registered).

## Background

Glaucoma is one of the leading causes of blindness worldwide, with an estimated prevalence of 2–4% in the 40 years and older age group [1, 2]. Glaucoma is a very heterogeneous group of diseases characterized by progressive atrophy of the optic disc caused by apoptotic retinal ganglion cell death [3, 4]. Known risk factors for the development of glaucoma are an elevated intraocular pressure (IOP), familial history of glaucoma, myopia and thin central corneal thickness [5–7]. Reduction of IOP by means of medication and/or surgery is the only known way to effectively slow down further disease progression [8, 9].

Usually, IOP reduction by means of medication is the first-line treatment in glaucoma therapy. Surgical intervention is indicated in cases of insufficient medical IOP reduction, problems with side effects, insufficient adherence and persistence to therapy and further progression of optic nerve damage [10].. Trabeculectomy (TE) with or without the use of antimetabolites was first introduced in the mid-1960s and has since remained the “gold standard” for long-term surgical IOP reduction [11, 12]. However, TE has numerous risks and unintended side effects that limit its use to advanced disease stages [13–16]. In recent years, various techniques have been developed to minimize surgical trauma and the risk of serious side effects. These newly developed techniques make use of different mechanisms to reduce the resistance of aqueous humor outflow, thereby reducing IOP. These new techniques are summarized under the term minimally invasive glaucoma surgery (MIGS) [17].

A promising MIGS procedure is the XEN microstent. The XEN microstent is approved for the treatment of primary open-angle glaucoma (POAG). The XEN microstent facilitates subconjunctival drainage of aqueous humor from the anterior chamber, which is comparable to the mode of action utilized in TE [18]. The XEN microstent does not comprise a valve mechanism, but maintains a minimum IOP of approximately 8 mmHg through its length and internal lumen [19].

XEN microstent studies reported in the literature are very heterogeneous in terms of design, glaucoma entity, inclusion and exclusion criteria, preoperative pressure and type of analysis. XEN microstent significantly reduced IOP and medication use as a solo or as a combined procedure in most glaucoma entities but number of patients are low in other than POAG. Significant differences in study results may be due to the way data have been analyzed or different statistical problems due to follow up loss [20].

However, subconjunctival drainage of aqueous humor is suitable for most types of glaucoma to lower or stop visual field progression rate. Therefore, the concept of this study was to analyze the IOP-lowering efficacy of the XEN microstent in the treatment also of other types of glaucoma. Other glaucoma entities suitable for XEN implantation were pseudoexfoliation glaucoma (PEX), secondary open-angle glaucoma of various causes (Sec.Gl) and normal tension glaucoma (NTG). The main objective of the investigations was to analyze whether or not the XEN implant is also suitable for the treatment of these types of glaucoma. Furthermore, all important parameters such as visual field progression, needling rate, changes in medication, complications and visual acuity development were to be investigated in all patients over the entire 12-month period, with 12-month data being available for all patients.

## Methods

The medical records of all patients/eyes scheduled for XEN microstent implantation from January 1, 2017 to December 31, 2018 at the University Eye Hospital in Leipzig were analyzed for this retrospective study.

The study was approved by the local ethics committee of the University Leipzig, Leipzig, Germany. Written informed consent was obtained from all patients. All procedures performed met the ethical standards of the institutional research commission as well as the Helsinki Declaration of 1964 and its later amendments. The trial was registered with the DRKS (registration number: DRKS00020800, Registered 25. February 2020 - Retrospectively registered,https://www.drks.de/drks_web/navigate.do?navigationId=trial. HTML&TRIAL_ID = DRKS00020800).

Signs of optic disc changes such as an increased cup/disc ratio, optic disc hemorrhages, nerve fiber layer defects and/or indentations of the papillary vessels verified glaucoma diagnosis. Untreated IOP had to be ≥21 mmHg in POAG, PEX and secondary glaucoma cases. In the cases analyzed, surgical intervention was necessary due to disease progression or medically uncontrollable IOP. In NTG IOP was by definition lower than 21 mmHg and disease progression alone was the single indication for surgery. This was demonstrated by deterioration of repeated automated standard perimetry results and/or reduction of retinal nerve fiber layer (RNFL) thickness on repeated optical coherence tomography (RNFL-OCT) examinations. Topical IOP-lowering therapy was discontinued 4 weeks prior to surgery in eyes scheduled for XEN implantation and preservative-free steroid eye drops were administered four times daily to achieve an irritation free conjunctiva. Additionally systemically acting acetazolamide (250 mg b.i.d.) was started 4 weeks before surgery to prevent IOP spikes. Exclusion criteria were a patient age below 40 years and a narrow anterior chamber angle. If both eyes of a single patient required surgical treatment for glaucoma, only the results of the first-operated eye were included into this analysis.

Four weeks prior to surgery, a ophthalmological examination was performed with anti-glaucomatous local medication. This included measurement of best-corrected visual acuity (BCVA) (Snellen charts converted to logMAR), objective refraction, slit-lamp examination with evaluation of the optic disc by indirect ophthalmoscopy and RNFL-OCT scans; Spectralis, Heidelberg Engineering, Heidelberg, Germany), automated standard perimetry (Twinfield 2, Oculus, Wetzlar, Germany; 24–2 test strategy, 55 targets), IOP measurement with Goldmann applanation tonometry and gonioscopy with the Sussmann four-mirror contact lens. The indication for the planned surgical procedure was re-evaluated, whereby the aim was to lower IOP and/or to reduce the number of necessary IOP-lowering agents. The demographic patient data collected prior to surgery were age, sex and laterality of the operated eye. The surgical procedure included only single XEN microstent implantation. None of the included cases were combined with phacoemulsification and IOL implantation.

### Surgical technique

The surgical technique used has already been described in previous publications of the group [21]. In short, two paracenteses were applied and the anterior chamber was filled with a dispersive viscoelastic agent. 0.1 ml 0.01% mitomycin C (MMC) was administered subconjunctivally. The XEN microstent injector was then passed through the sclera ab interno and excised subconjunctivally. The XEN stent was then implanted. Where necessary, the stent tip was straightened with a 30G needle as part of a primary needling procedure.

Postoperative visits were scheduled at day 1 and 2 as well as at month 1, 3 and 6 and 12 after surgery. All patients were examined at all examination dates. All local and oral antiglaucoma medications were discontinued. Preservative-free steroid eye drops were given 6 times daily for 4 weeks and then slowly reduced over 3 months. In cases with suspected steroid-induced IOP increase, which was usually encountered at the follow-up visit 1 month after surgery, steroids were reduced faster than usual over the next 3 weeks and additional IOP-lowering agents were applied. IOP lowering compounds used were usually adapted to the therapy given before surgery when tolerated. If IOP increase was due to conjunctival scaring with absence of a visible bleb / conjunctival filtration zone at any time point during follow-up a needling procedure was scheduled. Additionally preservative-free antibiotic eye drops were prescribed four times daily for 1 week. At each visit BCVA and IOP were measured and the anterior and posterior eye segments were evaluated for adverse events (conjunctival scarring, flat bleb, shallow anterior chamber, choroidal detachment, hypotony maculopathy etc.). The number of glaucoma medications taken was queried. In addition, visual field examinations were performed during the visits 6 and 12 months after the surgery.

For additional necessary post-surgical needling procedures, a 27G cannula was used on a syringe with 5-Fluoruracil (5-FU) (5 mg in 0.1 ml). The needle was inserted under the conjunctiva 3 mm temporal from the XEN stent and needling was performed by sweeping the needle tip posteriorly above and beneath the XEN microstent. Finally 0.1 ml of 5-FU was injected posterior to the end of the XEN microstent.

Clinical success was defined according to the recommendations of the World Glaucoma Association [22]. For complete success (A) an IOP reduction of ≥20% from baseline and resulting IOP of < 21 mmHg without the use of IOP-lowering drugs had to be achieved. For qualified success (A) the same criteria applied as for complete success A with the use of additional IOP-lowering drugs permitted. For complete success (B) an IOP reduction of ≥30% from baseline and resulting IOP of < 18 mmHg without the use of IOP-lowering drugs had to be achieved. For qualified success (B) the same criteria applied as for complete success B with the use of additional IOP-lowering drugs permitted. For complete success (C) an IOP reduction of ≥40% from baseline and resulting IOP of < 15 mmHg without the use of IOP-lowering drugs had to be achieved. For qualified success (C) the same criteria applied as for complete success C with the use of additional IOP-lowering drugs permitted.

Data collection and statistical analysis were performed using Excel (Version 2007, Microsoft; Redmond, USA) and SPSS (IBM Version 22.0; Chicago, Illinois, USA). The tested indices for patient age, BCVA, objective refraction and visual field are given as mean and standard deviation. The differences between the pre- and postoperative results for IOP, IOP reduction, BCVA, number of active IOP-lowering agents applied and the mean defect of static automated perimetry were analyzed using the Kruskal-Wallis nonparametric test or the Mann-Whitney test (where applicable) for intergroup comparisons and the Friedman test for intragroup comparisons of results measured in groups of different glaucoma types. A *p* ≤ 0.05 indicated statistical significance.

## Results

For this analysis the clinical course of 153 eyes of 153 patients (80 female and 73 male; 77 right and 76 left eyes) were included. Four groups of eyes suffering from different types of glaucoma were treated. 113 eyes (74%) were treated for POAG, 5 eyes (3%) for NTG, 13 eyes (9%) for secondary glaucoma and 22 eyes (14%) for pseudoexfoliation glaucoma. Of the 13 secondary glaucoma cases, four were due to previous eye trauma, four were due to neovascularization (2 cases of proliferative diabetic eye disease and 2 cases of central retinal vein occlusion, the underlying disease being considered in a stable phase at the time of XEN implantation), three cases were secondary to uveitis and the last two cases were due to steroid-induced glaucoma. In the complete patient group the mean age of patients at the time of surgery was 70.2 ± 10.8 years. The difference in mean patient age was not statistically significant between the four treated patient groups (*p* = 0.24). Before surgery the mean IOP of the 153 eyes was 23.9 ± 7.4 mmHg in the 153 eyes. Statistical analysis revealed significant IOP differences between the four subgroups (Kruskal-Wallis: *p* < 0.01). Further analysis revealed differences of statistical significance when comparing IOP results between POAG and PEX groups (*p* = 0.001), NTG and PEX groups (*p =* 0.001) and NTG and secondary glaucoma groups (*p* = 0.02). The mean number of active substances contained in the applied IOP-lowering eye drops was 2.6 ± 1.2 before surgery, and the comparison between the four treated groups showed no statistically significant difference (Kruskal-Wallis: *p* = 0.56). The mean defect tested by standard automated perimetry was 10.1 ± 4.3 dB before surgery, and the comparison between the four treated groups showed no statistically significant difference (Kruskal-Wallis: *p* = 0.96). The exact demographic data are also presented in Table 1.

**Table 1:** Patient demographic data

**age [years]**	**70.2 ± 10.8** (*p* = 0.24) *POAG*: 70.5 ± 8.5 *NTG*: 76.6 ± 3.9 *PEX*: 73.9 ± 5.9 *Secondary Glaucoma*: 68.7 ± 10.7
**laterality**	77 right; 76 left
**gender**	80 female; 73 male
**diagnosis**	POAG: 113 eyes (74%) NTG: 5 eyes (3%) PEX: 22 eyes (14%) Secondary glaucoma: 13 eyes (9%)
**number of medications [n]**	**2.6 ± 1.2** (*p* = 0.56) *POAG*: 2.5 ± 1.2 *NTG*: 2.6 ± 0.9 *PEX*: 2.9 ± 1.2 *Secondary Glaucoma*: 2.7 ± 0.9
**mean deviation [dB]**	**10.1 ± 4.3** (*p =* 0.96) *POAG*: 10.2 ± 3.9 *NTG*: 10.8 ± 2.9 *PEX*: 9.7 ± 4.7 *Secondary Glaucoma*: 10.1 ± 6.8

Patient demographic data for the 153 eyes treated with XEN microstent implantation in uncontrolled glaucoma. Also shown are the glaucoma types and the percentage of each glaucoma type as a proportion of the total treated eye group

### IOP results

**Fig. 1:**
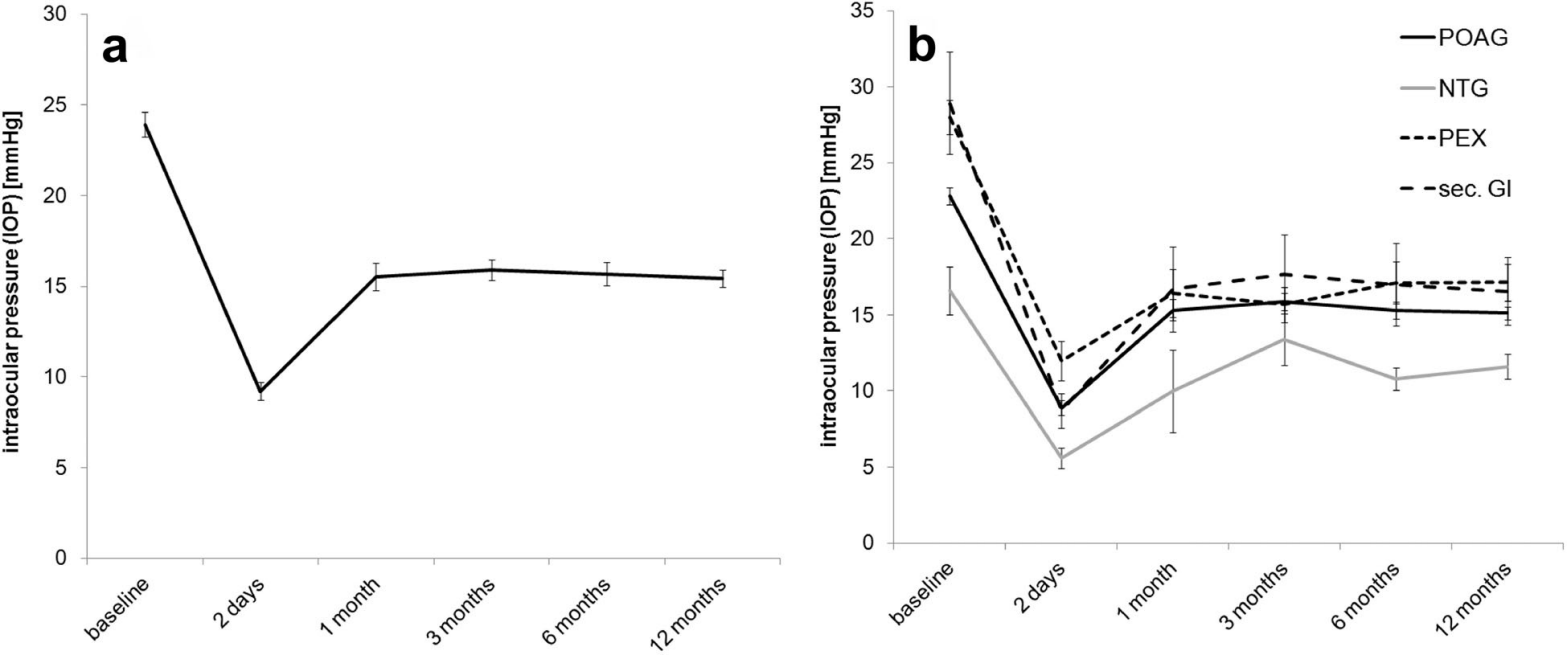
Mean IOP over study time. Course of mean IOP (in mmHg) for the whole group (**a**) as well as the subgroups (**b**) according to glaucoma type, i.e. primary open angle glaucoma (POAG), normal pressure glaucoma (NTG), pseudoexfoliation glaucoma (PEX) and secondary glaucoma of different etiology (see eq.)

**Fig. 2:**
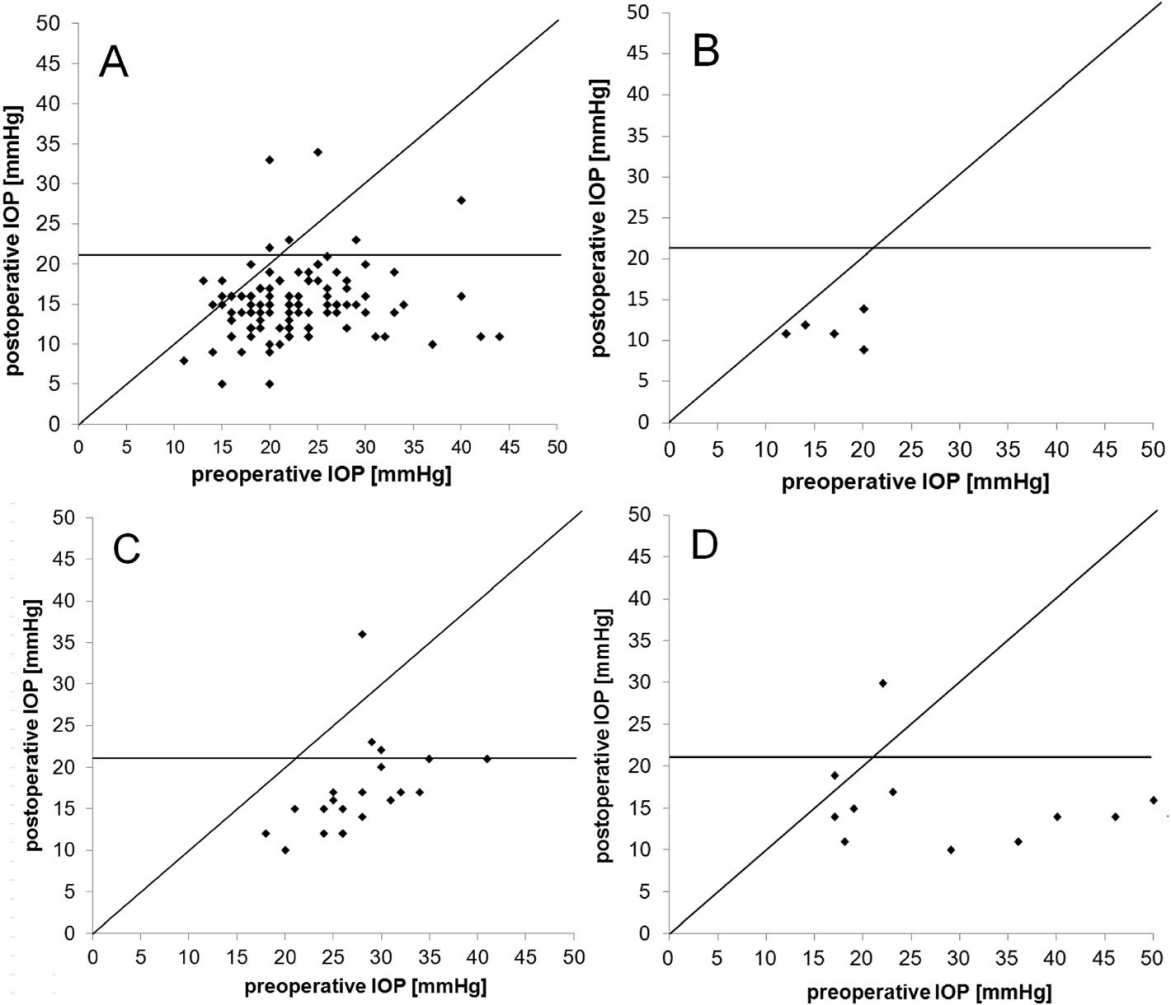
Pre- and postoperative IOP in different groups of glaucoma. Scattergram of pre- and 12-month postoperative IOP (in mmHg) in the four subgroups of glaucoma treated with XEN-microstent-implantation in (**a**) primary open angle glaucoma, (**b**) normal pressure glaucoma, (**c**) pseudoexfoliation glaucoma, (**d**) secondary glaucoma. The 21 mmHg-value is highlighted with a horizontal line to recognize the proportion of cases with an IOP below or above 21 mmHg 12 months after surgery

In the complete group of eyes analyzed mean IOP before surgery was 23.9 ± 7.4 mmHg. Two days after XEN implantation, mean IOP decreased to 9.2 ± 5.2 mmHg and then increased again to 15.5 ± 7.9 mmHg 1 month and to 15.9 ± 6.1 mmHg 3 months after surgery. Thereafter, mean IOP remained stable with a mean value of 15.4 ± 5.1 mmHg 1 year after XEN implantation, which corresponds to a mean IOP reduction of 31% compared to baseline before surgery. For the complete group of eyes analyzed the difference between the mean IOP results measured at all follow-up examinations and those measured at baseline was statistically significant at all-time points (*p* < 0.01). The exact values and the course of mean IOP during the first 12 months after surgery for the complete group as well as for the four treated subgroups are summarized in Figs. 1 & 2 and Table 2**.**

**Table 2:** Mean IOP in different groups of glaucoma at all examination time points

	baseline	2 days	1 month	3 months	6 months	12 months	***p***=	mean reduction [%]
**complete group**	23.9 ± 7.4	9.2 ± 5.2	15.5 ± 7.9	15.9 ± 6.1	15.7 ± 6.7	15.4 ± 5.1	**< 0.01**	−31.3 ± 23.9
**POAG**	22.8 ± 6.5	8.9 ± 5.1	15.3 ± 7.6	15.9 ± 6.0	15.3 ± 6.2	15.1 ± 4.6	**< 0.01**	−29.8 ± 23.8
**NTG**	16.6 ± 3.4	5.6 ± 2.7	10.0 ± 3.0	13.4 ± 2.9	10.8 ± 4.6	11.6 ± 2.2	**< 0.05**	−28.6 ± 18.4
**PEX**	28.0 ± 7.9	11.9 ± 6.4	16.5 ± 8.0	15.7 ± 6.2	17.1 ± 7.2	17.1 ± 6.6	**< 0.01**	−37.8 ± 17.9
**secondary glaucoma**	28.9 ± 13.9	8.7 ± 4.6	16.7 ± 10.6	17.7 ± 10.3	17.0 ± 10.4	15.5 ± 6.9	**< 0.05**	−35.7 ± 36.3

Mean IOP (mmHg) measured at baseline and all follow-ups after XEN microstent-implantation. Results are reported for the whole group of eyes treated and for the four subgroups of eyes with different glaucoma types. The indicated *P*-values and mean reduction represent the comparison between the results of the baseline examination and the 12-month results of the respective group

### IOP results in different types of glaucoma

Mean IOP at baseline before XEN implantation in the 113 POAG eyes was 22.8 ± 6.5 mmHg. Two days after surgery mean IOP dropped to 8.9 ± 5.1 mmHg. From the follow-up examination 1 month after surgery the IOP remained stable at about 15 mmHg and 1 year postoperatively the mean value was 15.1 ± 4.6 mmHg. This corresponds to an average IOP decrease of 31% compared to baseline. Comparison of mean IOP results before and after surgery showed a difference of statistical significance (*p* < 0.01) at all follow-up examinations.

In the 5 NTG eyes the mean IOP before XEN implantation was 16.6 ± 3.4 mmHg. Mean IOP decreased to 5.6 ± 2.7 mmHg 2 days after surgery and rose again to 10.0 ± 3.0 mmHg 1 month after surgery and remained relatively stable thereafter with a mean value of 11.6 ± 2.2 mmHg 12 months after surgery. This corresponds to an IOP reduction of 29% from baseline. IOP was well regulated, with all five eyes achieving an IOP < 15 mmHg 12 months after surgery. Comparison of mean IOP results before and after surgery showed a difference of statistical significance (*p* < 0.05) at all follow-up examinations.

In the 22 treated eyes suffering from PEX mean IOP before surgery was 28.0 ± 7.9 mmHg. Similarly to the other treatment groups mean IOP decreased to 11.9 ± 6.4 mmHg 2 days after surgery and increased to 16.5 ± 8.0 mmHg 1 month after surgery. Thereafter, IOP remained reasonably stable with a mean value of 17.1 ± 6.6 mmHg 1 year after XEN implantation, which corresponds to a decrease of 38% compared to baseline. The comparison between the average IOP results before and after surgery showed a difference of statistical significance (*p* < 0.01) at all follow-up examinations.

In the 13 eyes suffering from secondary glaucoma mean IOP before surgery was 28.9 ± 13.9 mmHg. Mean IOP dropped to 8.7 ± 4.6 mmHg 2 days after surgery. Thereafter, as in the other treatment groups, mean IOP increased to 16.7 ± 10.6 mmHg 1 month after XEN implantation and remained stable with a mean of 15.5 ± 6.9 mmHg at the follow-up examination 1 year after surgery, which corresponds to a mean IOP decrease of 36% compared to baseline. Comparison of follow-up to baseline results again showed a difference of statistical significance at all-time points (*p* < 0.05).

Further comparison of IOP results between the four treatment groups 12 months after XEN microstent implantation also revealed differences of statistical significance (Kruskal-Wallis: *p* < 0.01). Here further testing revealed differences of statistical significance between POAG and NTG groups (*p* = 0.029) as well as between NTG and PEX groups (*p* = 0.006). However, when comparing results for mean IOP reduction from baseline to 12 months after surgery between the four groups, no difference of statistical significance was found (Kruskal-Wallis: *p* = 0.2).

### Complete and qualified success

Taking into account the WGA guidelines and the criteria summarized above defining complete and qualified success levels A to C, in the complete group of 153 eyes 60% achieved a complete success (A) and 74% a qualified success (A). Complete success levels B and C were achieved by 51 and 45% respectively. Qualified success B and C was achieved by 61 and 56% in the complete group of 153 eyes operated. The exact results for complete and qualified success levels A to C achieved in the four subgroups of different glaucoma entities is summarized in Table 3. Further analysis revealed differences of statistical significance between the four groups treated only concerning complete success (A) (Kruskal-Wallis: *p* = 0.03) and qualified success (C) (Kruskal-Wallis: *p* = 0.01). Further testing revealed that the aforementioned differences of statistical significance were due to higher success rates in the PEX group than in the POAG group (Mann-Whitney test: POAG~PEX for complete success (A) *p* = 0.004 and POAG~PEX for qualified success (C) *p* = 0.001).

**Table 3:** Clinical success rates for different groups of glaucoma

	Complete success (A)	Qualified success (A)	Complete success (B)	Qualified success (B)	Complete success (C)	Qualified success (C)
**complete group**	60%	74%	51%	61%	45%	56%
**POAG**	54%	71%	45%	57%	29%	38%
**NTG**	60%	60%	60%	60%	20%	20%
**PEX**	86%	91%	77%	82%	50%	59%
**secondary glaucoma**	75%	75%	58%	58%	50%	50%
**Kruskal-Wallis test**	***p =*** **0.03**	*p =* 0.22	*p* = 0.07	*p* = 0.17	*p =* 0.17	***p =*** **0.01**

Clinical Success defined as complete (without the use of IOP-lowering drugs) and qualified (with the use of additional IOP-lowering drugs) success for the complete treated group of eyes and the four subgroups of the different glaucoma types (A) IOP reduction of ≥20% from baseline and resulting IOP of < 21 mmHg; complete success (B) IOP reduction of ≥30% from baseline and resulting IOP of < 18 mmHg; complete success (C) IOP reduction of ≥40% from baseline and resulting IOP of < 15 mmHg). Additionally, distribution of achieved success levels was compared between treatment groups using Kruskal-Wallis test

### IOP-lowering medication

**Fig. 3:**
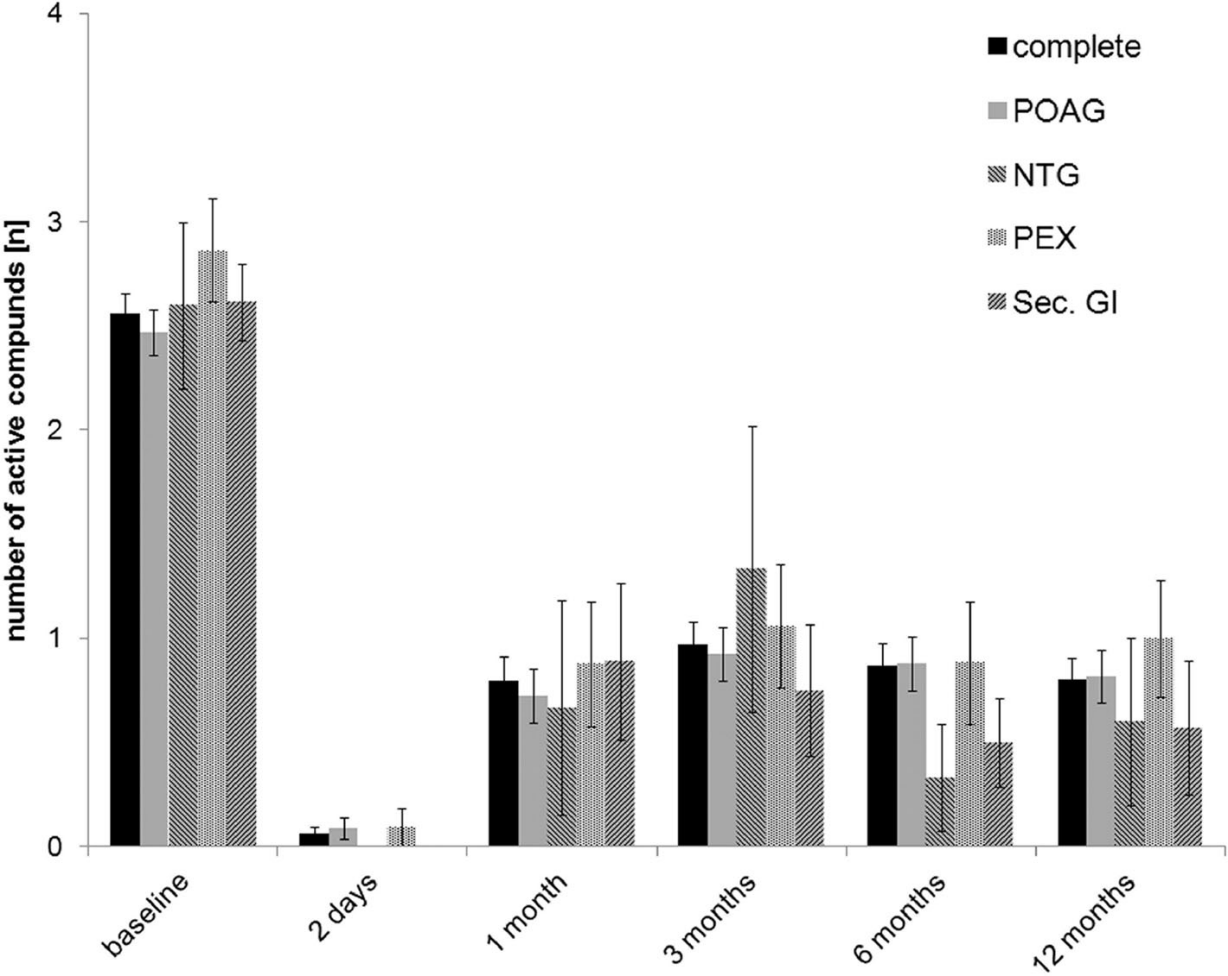
IOP-lowering agents over study time. Progression of the number of mean IOP-lowering agents (number) used in the entire XEN Microstent patient group and in the subgroups depending on glaucoma type during the first 12 months of postoperative follow-up. (primary open angle glaucoma (POAG); normal pressure glaucoma (NTG); pseudoexfoliation glaucoma (PEX); secondary glaucoma (sec. Gl))

The exact course of the number of IOP-lowering eye drops applied is summarized in Fig. 3 and Table 4. The average number of active substances applied in the entire group of 153 eyes fell from 2.6 ± 1.2 before to 0.8 ± 1.3 12 months after XEN implantation (*p* < 0.01). In the four subgroups treated the number of applied drugs decreased from 2.5 ± 1.2 to 0.8 ± 1.3 in the POAG group (*p <* 0.01), from 2.6 ± 0.9 to 0.6 ± 0.9 in the NTG group (*p* < 0.05), from 2.9 ± 1.2 to 1.0 ± 1.3 in the PEX group (*p <* 0.01) and from 2.7 ± 0.9 to 0.7 ± 1.4 in the group of eyes suffering from secondary glaucoma (*p <* 0.01) during the first 12 months after surgery.

**Table 4:** Mean IOP lowering agents in different groups of glaucoma at all examination time points

	baseline	2 days	1 month	3 months	6 months	12 months	***p***=
**complete group**	2.6 ± 1.2	0.1 ± 0.5	0.8 ± 1.4	1.0 ± 1.4	0.9 ± 1.4	0.8 ± 1.3	**< 0.01**
**POAG**	2.5 ± 1.2	0.1 ± 0.5	0.7 ± 1.4	0.9 ± 1.4	0.9 ± 1.4	0.8 ± 1.3	**< 0.01**
**NTG**	2.6 ± 0.9	0 ± 0	0.7 ± 1.2	0.7 ± 1.0	0.3 ± 0.6	0.6 ± 0.9	**< 0.05**
**PEX**	2.9 ± 1.2	0.1 ± 0.4	0.9 ± 1.4	1.1 ± 1.4	0.9 ± 1.4	1.0 ± 1.3	**< 0.01**
**secondary glaucoma**	2.6 ± 0.9	0 ± 0	0.9 ± 1.8	0.8 ± 1.5	0.5 ± 1.0	0.6 ± 1.5	**< 0.01**

Examination values for the number of applied IOP-lowering agents at the beginning of the treatment as well as for all follow-ups after XEN implantation for the whole treated group of eyes and the four subgroups of the different glaucoma types. The *P-*values given represent the comparison between the baseline and 12-month results for each group

### Visual acuity

**Fig. 4:**
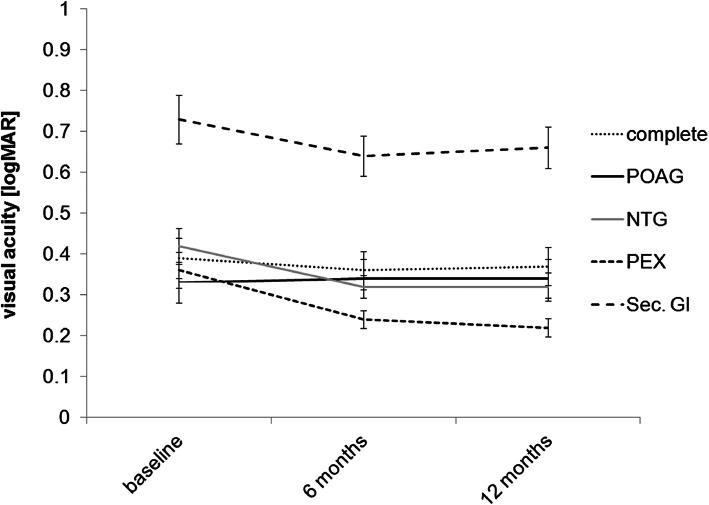
Visual acuity over study time in different groups of glaucoma. Course of BCVA in the entire group of eyes treated with XEN microstent implantation and in subgroups depending on the type of glaucoma in the first 12 months of postoperative follow-up.(primary open angle glaucoma (POAG); normal pressure glaucoma (NTG); pseudoexfoliation glaucoma (PEX); secondary glaucoma (sec. Gl))

In the entire group of 153 eyes treated with the XEN microstent, mean BCVA remained stable and showed no difference of statistical significance during postoperative follow-up. Mean BCVA was 0.38 ± 0.52 logMAR before surgery and 0.36 ± 0.49 logMAR 6 months and 0.37 ± 0.49 logMAR 12 months after surgery (*p* = 0.97 after 6 months and *p* = 0.88 after 12 months compared to baseline) (See also Fig. 4 for the exact course).

In the 113 POAG eyes mean BCVA was 0.33 ± 0.47 logMAR before and 0.34 ± 0.47 logMAR 12 months after surgery (*p* = 0.31). This included 7 eyes with a BCVA loss of ≥0.2 lines during the 12 months of follow-up. Reasons for this loss of function was glaucoma disease progression in 2 cases, AMD progression in 3 cases, CSME development in 1 case and further progression of senile cataract in 1 case. In the 5 NTG eyes mean BCVA was 0.42 ± 0.46 logMAR before and 0.36 ± 0.37 logMAR 12 months after surgery (*p* = 0.22). In the NTG group no case of BCVA loss of ≥0.2 lines was registered during follow-up. In the 13 PEX eyes mean BCVA was 0.36 ± 0.46 logMAR before and 0.22 ± 0.23 logMAR 12 months after surgery (*p* = 0.92). In the PEX group one case of vision loss of ≥0.2 lines was registered, which was due to progression of glaucoma. In the group of eyes treated for secondary glaucoma mean BCVA was 0.74 ± 0.63 logMAR before and 0.66 ± 0.53 logMAR 12 months after surgery (*p* = 0.91). This included one case experiencing vision loss of ≥0.2 lines, which was due to progression of glaucoma.

Comparison of BCVA between groups showed that there were differences of statistical significance concerning BCVA (Kruskal-Wallis: *p* < 0.01). Mean BCVA was worse in the secondary glaucoma group than in the three other groups treated. Further performed Mann-Whitney tests confirmed a difference of statistical significance when comparing BCVA between the secondary glaucoma and the POAG groups (*p <* 0.01) and between the secondary glaucoma and the PEX groups (*p <* 0.01). Further testing did not reveal differences of statistical significance between any of the other groups. Finally, when comparing the BCVA change occurring over the 12 month follow-up period in between the four groups treated no difference of statistical significance could be revealed (Kruskall-Wallis; *p* = 0.35).

### Visual field

Simultaneously with the drop of mean IOP, mean visual field defect remained stable. In the complete group of 153 eyes treated mean defect was 10.1 ± 4.3 dB at baseline, increased slightly to 10.8 ± 4.3 dB at 6 months (*p* = 0.11) and was 10.8 ± 4.2 dB at 12 months after surgery, which was not statistically significant (*p* = 0.15). Also, the comparison of mean BCVA between the four treatment groups before and 12 months after surgery did not reveal differences of statistical significance (Kruskal-Wallis; before surgery: *p* = 0.08; 12 months: *p =* 0.3) (see Table 5 for details).

**Table 5:** Visual field defects at different time points in different groups of glaucoma

	baseline	6 months	12 months	***p***=
**complete group**	10.1 ± 4.3	10.8 ± 4.3	10.8 ± 4.2	0.11
**POAG**	10.2 ± 3.9	10.6 ± 3.9	10.5 ± 3.9	0.08
**NTG**	10.9 ± 2.9	10.8 ± 2.3	10.8 ± 2.5	0.23
**PEX**	9.7 ± 4.7	9.8 ± 4.5	9.6 ± 4.7	0.17
**secondary glaucoma**	10.0 ± 4.9	10.7 ± 5.0	10.7 ± 5.0	0.37

Examination values for mean deviation (in decibels) of automated standard perimetry at baseline and follow-up 6 and 12 months after XEN implantation for the entire patient population and the four subgroups of different glaucoma types. The *P-*values given represent the comparison between the results at baseline and at 12 months for each group and do not show any statistically significant difference for any of the groups analyzed

### Complications and needling rate

To achieve the results described, 64 needling procedures had to be performed in 54 of the 153 eyes (35.3%). 44 eyes needed 1 needling and another 10 eyes needed 2 needlings during the first year after XEN implantation. None of the eyes needed > 2 needlings. The needling rate was different between the four subgroups analyzed (see Table 6). The lowest needling rate was found in NTG eyes (20%) and the highest rate in PEX glaucoma eyes (55%). In the total group of 153 eyes treated 20 eyes showed benign choroidal detachment during the first postoperative month. All cases responded well to conservative treatment (contact lens and cycloplegia). A large and prominent subconjunctival hemorrhage (to the extent that it was classified as clinically significant) was seen in 12 cases immediately after surgery, but was resorbed after 1 to 4 weeks. Blood in the anterior chamber immediately after surgery was seen in 10 eyes and dissolved in all cases without further consequences after 1 to 4 weeks. Postoperative macular edema was seen in 3 eyes, which all responded well to treatment with carbonic anhydrase inhibitors. Apart from the above described incidents, no serious complications were observed in the treated eyes.

**Table 6:** Needling rates and complications

needling procedures	64 in 54 eyes POAG: 38 of 113 (34%) NTG: 1 of 5 (20%) PEX: 12 of 22 (55%) Sec. Gl.: 3 of 13 (23%)
choroidal detachment	20 of 153 (13%)
prominent or large subconjunctival hemorrhage	12 of 153 (8%)
hyphaema	10 of 153 (7%)
shallow anterior chamber	3 of 153 (2%)
macular edema	3 of 153% (2%)
conjunctival erosion	2 of 153 (1%)
uveitis	1 of 153 (< 1%)
keratitis	1 of 153 (< 1%)
corneal erosion	1 of 153 (< 1%)

Needling rate for the whole group of patients (as well as for the four subgroups of the different types of glaucoma treated) and the rate of other complications observed during the first 12 months of follow-up after XEN microstent implantation in uncontrolled glaucoma in all groups together

## Discussion

Classic TE with or without the use of antimetabolites is the “gold standard” for surgical treatment of a wide range of glaucoma entities. The XEN microstent is designed to bypass the transtrabecular outflow pathway whereby reducing IOP. Since its initial description, a number of reports have been published on the efficacy and safety profile of the XEN microstent for the treatment of POAG [18, 23, 24]. In summary, implantation of the XEN microstent can significantly reduce IOP and the amount of IOP-lowering eye drops applied, resulting in acceptable success rates in POAG after 12 and 24 months. Reitsamer and colleagues already showed in their one and two-year multicenter study (APEX study) that IOP and required medication can be reduced effectively by implantation of the XEN microstent either alone or in combination with phacoemulsification and posterior chamber lens implantation [23] Some groups even reported on achieving lower mean postoperative IOP values of around 12 mmHg when starting from a lower initial mean level at baseline before surgery [25].

Grover et al. reported on their 12-month single XEN procedure results in a group of 65 eyes with uncontrolled and already pre-operated glaucoma cases (85% of eyes had already been unsuccessfully treated with filtering surgery and/or cilioablative techniques) [26] The results were similar to the data hereby reported with a qualified success rate of 75%, a reduction of mean IOP from 25.1 ± 3.7 mmHg at baseline to 15.9 ± 5.2 mmHg 12 months after surgery and a needling rate of about 32%. The patient cohort reported on also included a large percentage of POAG cases and some PEX cases, but no case of secondary glaucoma. Grover et al. also did not report on the efficacy of the XEN microstent in subgroups of different glaucoma entities. Lenzhofer et al. already demonstrated the long-term effectiveness of the XEN microstent in a group of 64 eyes of 64 POAG patients. It was shown that low mean IOP values and reduced necessity for application of IOP-lowering medication can even be found as long as 4 years after implantation of the XEN microstent [27]. However, these data were collected in eyes in which the XEN 63 was implanted (with a larger internal lumen of 63 μm). Recently Fea et al. reported a prospective multicenter study with different types of glaucoma in 115 patients, 56 patients got combined surgery with phacoemulsification and the follow up was 12 months [28]. Mean IOP and medications decreased significantly until study end point. An IOP reduction of 20% was achieved in 72,3 and 30% reduction in 52.6%.

Given the mode of action utilized, the XEN microstent should in theory also be applicable in glaucoma entities other than POAG. Although some groups reported on the treatment of different types of glaucoma with the XEN microstent, they so far failed analyzing the efficacy in the treated subgroups [24, 29]. We analyzed the 12-month postoperative follow-up results in NTG, PEX and secondary glaucoma cases and compared the results with those obtained in a medium-sized comparison group of POAG eyes. With the reported postoperative results we could clearly and for the first time demonstrate that IOP and necessary IOP-lowering medication can be lowered with a comparable efficacy in NTG, PEX and secondary glaucoma cases as in POAG. Apart from this visual acuity and visual field indices remained stable without evidence for further postoperative deterioration, although only the first 12 months after surgery were monitored. There was a notable difference in mean BCVA, which was worse but also stable during the postoperative course in the subgroup of treated secondary glaucoma cases. This must be interpreted within the context of the underlying disease process leading to secondary glaucoma, which in most cases has devastating consequences on visual acuity.

Postoperative results after implantation of the XEN microstent other types of glaucoma than POAG have also been described by other authors. Ibanez-Munoz and colleagues implanted the XEN microstent in 36 eyes with POAG and 37 eyes with secondary glaucoma, 34 of which had PEX glaucoma [30]. The difference in the postoperative results regarding the reduction of IOP and the required medication was not statistically significant in both groups. Apart from that, no difference was found when the XEN microstent was implanted as a solo procedure or in combination with cataract extraction and IOL implantation.

Publications on study data for 1 year and longer from single XEN microstent implantations without phacoemulsification are rare. Most studies combine groups with and without phacoemulsification. The work from Grover et al. was already shown [26]. Tan et al. presented a retrospective interventinal case series of 39 eyes, some with previous glaucoma surgery [31]. With smaller different glaucoma groups than this study eyes showed in mean an IOP reduction from 24.9 to 14.5 mmHg with a decrease from 3 medications pre-surgery to 0,7 at 12 months. Complete Success was achieved in 87% (definition A) and 62% (definition B) while qualified success was 92 and 64% respectively.

Karimi et al. (like our group) have implanted the XEN microstent in different glaucoma types, including traumatic and neovascular glaucoma, but have not reported on postoperative results in these treated subgroups [32]. They also found no statistically significant differences in their results regarding IOP reduction and medication in cases where the XEN microstent was implanted alone or in combination with cataract extraction. In the 259 eyes treated, 41% required needling during the first 18 months after implantation. However, it should be noted that results originating from only 34 and 12% of eyes were included for analysis of the 12- and 18-month postoperative follow-up.

The higher rate of needling after XEN microstent implantation compared to TE, the “gold standard” in glaucoma surgery, has been the subject of criticism in the past. Schlenker et al. and others reported on further necessary secondary interventions and safety problems when comparing the results following both XEN microstent implantation and TE ± MMC, which showed a slightly higher needling rate after XEN, but which did not reach statistical significance during further analysis [33]. However, the different rates of necessary postoperative interventions and the rates of postoperative complications were not statistically significant between the groups treated with XEN microstent or TE. In summary, XEN microstent and TE are comparable in terms of postoperative safety. However TE led to more visits, especially in the first 3 months after surgery. As shown in our study PEX eyes had the highest needling rates but also the highest success rates. This was also confirmed in several studies so far as shown in the comprehensive recent review from Fea et al. [20] Success rates for XEN microstent as single procedure in uveitic glaucoma showed good results in a small number of patients in several studies from up to 60 to 65% reduction of IOP and significant medication lowering. Comparison for NTG are missing [20].

The main limitation of this study is that although the number of patients with other forms of glaucoma is comparatively small. This is usually the case in most studies because of the lower incidence of these glaucoma entities. Only eyes suffering from a medium to high glaucoma damage were included in this study. Further studies should focus on early stages of glaucoma, since the minimally invasive approach of MIGS would be interesting for early surgical intervention. Lowering IOP to mean values around 15 mmHg on average certainly represents significant reduction, but according to the advanced glaucoma intervention study (AGIS) is certainly no guarantee to prevent progression [34]. Lowering intraocular pressure to less than 12 mmHg would be necessary here and was not achieved in all eyes. An additional reduction of intraocular pressure by medication or surgery has to be discussed in case of further disease progression.

Contrary to many other studies, this analysis shows a high number of patients outside the classic POAG spectrum. In addition, all patients in this study were followed up exclusively with XEN Micro Stent Implantation as a single surgical procedure with detailed follow-up of various glaucoma parameters over 12 months. In this analysis only one eye per patient was included (even if both eyes were treated). And for all included eyes a full 12-month postoperative follow-up was available. One limiting factor, concerning the hereby presented data, is that the sizes of the four treatment groups were different. However, the largest group, which comprises POAG patients, should only be regarded as a comparison group for the other, less frequent glaucoma entities, treated under the same conditions. In the entire study group, XEN microstent implantation not only resulted in a reduction of IOP and necessary IOP-lowering drugs, but also had no significant effect on BCVA, neither in the total group of 153 eyes nor in the four subgroups analyzed. Lenzhofer et al. showed similar results in their treated POAG cases [35]. It has also been shown earlier that BCVA in eyes after TE decreases, even when taking into account (and ruling out) the known side effect of cataract formation [36].. Therefore, our hereby presented data also complement the growing evidence in favor for the XEN microstent in POAG and other types of glaucoma with an unblocked anterior chamber angle.

## Conclusion

Implantation of the XEN microstent was effective in reducing intraocular pressure and the number of drugs applied to reduce intraocular pressure, while BCVA and perimetry results remained unchanged. In conclusion, the data presented here on the efficacy and safety of the XEN microstent in glaucoma types other than POAG are encouraging for the use of this (comparatively new) technique also in the treatment of PEX and other secondary glaucoma cases. Longer follow-up time, a larger number of eyes treated and a comparison group comparing the efficacy of the XEN microstent to the “gold standard” of glaucoma surgery, which remains the TE, are necessary to further demonstrate the usefulness of this technique.

## Data Availability

The datasets obtained during and/or analyzed during the current study available from the corresponding author on reasonable request.

## Abbreviations

5-FU: 5-Fluoruracil
BCVA: Best corrected visual acuity
IOP: Intraocular pressure
MD: Mean deviation
MIGS: Minimally invasive glaucoma surgery
MMC: Mitomycin C
NTG: Normal-tension glaucoma
PEX: Pseudoexfoliation glaucoma
POAG: Primary open angle glaucoma
RNFL: Retinal nerve fiber layer
RNFL-OCT: Retinal nerve fiber layer thickness - optical coherence tomography
Sec.Gl: Secondary glaucoma
TE: Trabeculectomy
